# Healthcare worker compliance with cervical cancer screening guidelines. An audit at district and regional level of care in the Pietermaritzburg Metropolitan area of KwaZulu-Natal

**DOI:** 10.4102/sajhivmed.v21i1.1104

**Published:** 2020-09-02

**Authors:** Mbali T. Makhubo, Thinagrin D. Naidoo

**Affiliations:** 1 Department of Obstetrics and Gynaecology, Nelson R. Mandela School of Medicine, University of KwaZulu-Natal, Durban, South Africa; 2 Department of Obstetrics and Gynaecology, Grey’s Hospital, Pietermaritzburg, South Africa; 3 Nelson R. Mandela School of Medicine, University of KwaZulu-Natal, Durban, South Africa

**Keywords:** questionnaire, Likert scale, healthcare worker, cervical cancer, HIV, guidelines

## Abstract

**Background:**

In South Africa (SA) there are screening guidelines for cervical cancer in women living with HIV (WLWH). To our knowledge there is lack of data concerning the knowledge of health care workers (HCWs) about cervical cancer screening guidelines before the initiation of antiretroviral therapy (ART) in WLWH.

**Objectives:**

To investigate the knowledge and familiarity of HCWs regarding cervical cancer screening guidelines in WLWH.

**Methods:**

A cross-sectional questionnaire-based study exploring compliance with cervical cancer screening guidelines before initiating ART was conducted with 85 HCWs in the antiretroviral (ARV) clinics of a district and regional hospitals in KwaZulu-Natal, SA. Data were analysed using Stata V13 and a p-value of ≤ 0.05 was considered statistically significant.

**Results:**

Eighty-five HCWs were included in the study. Health care workers’ responses to knowledge about cervical cancer screening in WLWH were suboptimal and revealed significant gaps. Most HCWs did not know the screening intervals of WLWH. Statistically significant associations were found between an HCW’s occupation and responses to the Likert scale questions.

**Conclusion:**

Although the majority of HCWs were familiar with cervical cancer screening guidelines in WLWH, the study highlights that there are deficiencies in both knowledge and practice. Creating awareness among HCWs regarding the current methods of cervical cancer screening is a necessary to reduce morbidity and mortality from cervical cancer in WLWH.

## Introduction

The ‘dual epidemics’ of the human immunodeficiency virus (HIV) and cervical cancer have been responsible for the premature deaths of thousands of women in resource-constrained countries (RCCs).^1^ Screening is a vital key to the early detection and prevention of cervical cancer. Given the enormous burden of HIV and human papillomavirus (HPV) in South Africa (SA), healthcare workers’ (HCWs) knowledge of cervical screening and the management of both cervical cancer and HIV is vital to ensure the survival and well-being of women living with HIV (WLWH). The risk of HPV infection is increased, as are precancerous cervical lesions (risk increased by 2–12 times), in WLWH versus HIV-uninfected women.^2^ Linking cervical screening to antiretroviral therapy(ART) treatment initiation in WLWH provides HCWs with a unique opportunity to diagnose and manage both viral infections. If followed properly, the screening guidelines will reduce the risk of cervical cancer in RCCs.^3^ Premalignant lesions are reportedly four to five times more likely in WLWH than in uninfected women.^4^ Furthermore, cervical cancer in South African WLWH presents earlier (up to 18 years earlier) than HIV-uninfected peers.^5^ Well-implemented national cervical screening guidelines and programmes significantly reduce the attributable morbidity and mortality of cervical cancer in WLWH and HIV-uninfected women.^6^

Cervical screening is most effective when undertaken within an organised programme. In RCCs, including SA, screening rates and subsequent follow-up care remain suboptimal. Screening rates as low as 1% have been reported.^7^ Screening coverage is crucial for effective prevention. The problem is compounded in RCCs by poor call-back systems, weak infrastructure and inadequately skilled staff. Indeed, it is unethical to offer screening without ensuring that follow-up and treatment services are available for all women. It is essential therefore that the HCWs with appropriate training and skills who provide these services are familiar with the screening guidelines. To our understanding, there is a lack of data on the knowledge of HCWs in this regard. This study aimed to investigate the knowledge and familiarity of HCWs with cervical cancer guidelines in WLWH before initiating ART.

## Methodology

This study was a cross-sectional questionnaire-based descriptive survey of 85 HCWs in ART clinics in the Pietermaritzburg Metropolitan area of KwaZulu-Natal province, SA. The study population was stratified by profession into three groups: interns, medical officers and professional nurses. The cohort was recruited from staff members attached to the antiretroviral (ARV) clinics of the Northdale (District) and Edendale (Regional) hospitals. The study participants were randomly selected from each group. A survey cover sheet explaining the purpose of the study was attached to the questionnaire and was signed by those who completed the questionnaire. To ensure anonymity and confidentiality, personal-subject identifiers were not used in the questionnaire itself. The principal investigator enrolled only participants who willingly and voluntarily filled in and returned the questionnaire.

## Data collection

A structured and pretested, self-administered questionnaire was used for data collection. The collected data were checked for completeness and consistency by the principal investigator and the supervisor. Concerning the guidelines, experts in research methodology, obstetrics and gynaecology, and oncology confirmed the validity of the questionnaire before the pilot study commenced. The instrument was pretested on 10 study participants working in other health facilities who were not part of the actual study. Findings from the pre-test were used to modify the instrument and clarify the questions. The questionnaire was divided into two parts: the first part dealt with the socio-demographic profile and professional status of the respondents and the second part dealt with knowledge about the practice of cervical cancer screening in WLWH before initiating ART. The questionnaire was written in English.

Healthcare workers’ responses were assessed by asking them to rate each of the following statements on a five-point Likert scale:

- All WLWH must have a yearly pap smear.
- A pap smear must be done at the age of 21 years irrespective of HIV status.
- A pap smear in WLWH is only done after the age of 30 years.
- Cervical cancer screening in WLWH can be done anytime.
- Cervical cancer screening in WLWH can only be done after 3 years of initiating ART.
- If the results come back with high-grade squamous intraepithelial lesion (HSIL), the patient must repeat the pap smear.
- If the results come back with persistent low-grade squamous intraepithelial lesion (LSIL), keep the patient at the clinic without any further action.
- The incidence of cervical cancer in our population is very high.

Healthcare workers were asked to choose one of the following options for each of the statements listed above: ‘strongly agree’, ‘agree’, ‘neither agree nor disagree’, ‘disagree’ or ‘strongly disagree’. For ease of interpreting and presenting results, responses for ‘strongly agree’ and ‘agree’ and for ‘disagree’ and ‘strongly disagree’ were combined. Participants’ practices were assessed by asking specific questions about practices regarding cervical cancer screening.

## Statistical analysis

A descriptive analysis of the HCW’s demographic characteristics is presented. Frequencies and percentages were used to summarise categorical variables, such as the type of HCWs. Frequency distributions of numeric variables, such as age and years of experience, were examined for normality and means or medians used as appropriate. The outcome of the study was a knowledge score composed of the sum of the responses of each HCW to the questions posed and measured on the Likert scale. Subgroups were assessed by age, type of HCW and experience (years) and were compared with responses to individual questions, and an overall score was determined using the Chi-square test. Data were analysed using Stata V13 and a *p*-value of < 0.05 was considered statistically significant.

### Ethical consideration

Ethical clearance was obtained from the Biomedical Research Ethics Committee, the University of Natal (BE: 572/18), Department of Health and the various institutions before commencing the study. All participants provided written informed consent before enrolment.

## Results

Eighty-five HCWs were interviewed and enrolled in the study.

### Demographic characteristics and professional standing of participants

The majority of the HCWs were 35 years old or younger (60%), women (71.8%) and black people (52.9%). Professional nurses and medical officers accounted for 41.2% and 22.1% of the HCWs, respectively. A total of 66% of the HCWs had less than 3 years’ experience in the initiation of ARV drugs and only 19 (22.4%) had formal training in HIV management. A total of 51.8% of the HCWs practised at a district hospital (Northdale), whereas the other half practised at the regional hospital (Edendale) (Table 1). Of the 19 HCWs who had formal training in HIV management, 7 (36.8%) had a diploma and 12 (62.2%) had a certificate in HIV management.

**TABLE 1:** Healthcare workers’ designation and experience or training in different levels of care.

Variables	*n*	%
**Category of HCWs**		
**Nursing staff**		
Professional nurses	35	41.2
**Medical practitioners**		
First-year medical intern	13	15.3
Second-year medical intern	16	18.8
Community service medical officer	1	1.2
Medical officer	19	22.4
Unknown	1	1.2
**Total**	**85**	**100**
**Number of years of experience in initiating antiretroviral drugs**		
< 3 years	56	65.9
3–5 years	17	20
> 5 years	12	14.1
**Total**	**85**	**100**
**Formal training in HIV management**		
Diploma in HIV management	7	8.2
Certificate in HIV management	12	14.1
No formal training in HIV management	66	77.6
**Total**	**85**	**100**
**Level of care of practice**		
Clinic	9	10.6
Hospital level 1	44	51.8
Hospital level 2	28	32.9
Hospital level 3	2	2.4
Unknown	2	2.4
**Total**	**85**	**100**

HIV, human immunodeficiency virus; HCWs, healthcare workers.

Of the 19 HCWs who had formal training in HIV management, 7 (36.8%) had a diploma and 12 (62.2%) had a certificate in HIV management.

### Awareness of cervical cancer screening guidelines and local protocols

Only two (2.4%) of the HCWs were aware of the KwaZulu-Natal cervical cancer screening guidelines, and 20 (23.5%) were aware of the World Health Organization (WHO) cervical cancer screening guidelines. Fifty-one (60%) of the HCWs considered that the guidelines on screening WLWH provided by their health facilities were adequate (Table 2).

**TABLE 2:** Knowledge of cervical cancer screening guidelines or protocol compiled by different institutions.

Cervical cancer and HIV guidelines	*n*	%
Yes, I am aware of the KwaZulu-Natal cervical cancer screening guidelines.	2	2.4
Yes, I am aware of the national cancer guidelines/cervical cancer screening guidelines.	17	20.0
Yes, I am aware of the cervical cancer screening guidelines in South Africa (SASOG 2015).	19	22.4
Yes, I am aware of the WHO cervical cancer screening guidelines.	20	23.5
Yes, I am aware of at least one of the above four guidelines.	57	67.1
Yes, I am aware of the uMgungundlovu district protocol cervical cancer screening.	23	27.1
Yes, my health facility is compliant with cervical cancer screening guidelines in WLWH.	51	60.10

HIV, human immunodeficiency virus; WHO, World Health Organization; WLWH, women living with HIV; South African Society of Obstetricians and Gynaecologists.

Twenty-three (27.1%) HCWs were aware of the uMgungundlovu district protocol on cervical cancer screening, of which 19 were professional nurses. Fifty-one (60.0%) HCWs felt that patients who had abnormal pap smear results should be referred for colposcopy (33 professional nurses and 17 medical officers), and 76 (89.4%) participants believed that the health facilities where they were currently working were compliant with the cervical cancer screening programme for HIV-positive patients. Only two (2.4%) participants (one first-year medical intern and one medical officer) were aware of more than one set of cervical cancer screening guidelines. The KwaZulu-Natal guidelines were the least known of all screening protocols.

### The knowledge and the implementation of cervical cancer screening guidelines

The knowledge of the respondents about cervical cancer screening in WLWH was assessed on a Likert scale. Healthcare workers’ responses to the various statements that were designed to test their knowledge and implementation of cervical cancer screening varied (see Table 3).

**TABLE 3:** Responses of healthcare workers to statements of cervical cancer in women living with human immunodeficiency virus (*n* = 85).†

Statements	Response	Agreed		Disagreed		Total	Response rate (%)
		*n*	%	*n*	%		
All WLWH must have a yearly pap smear	Disagree	66	80.5	16	19.5	82/85	96.5
A pap smear must be done at the age of 21 years irrespective of HIV status	Disagree	25	33.8	49	66.2	74/85	87.1
A pap smear in WLWH is only done after the age of 30 years	Disagree	8	10.4	69	89.6	77/85	90.6
Cervical cancer screening in WLWH can be done anytime	Agree	55	78.6	15	21.4	70/85	82.4
Cervical cancer screening in WLWH can only be done after 3 years of initiating ART	Disagree	2	2.4	81	97.6	83/85	91.6
If the results come back with HSIL, the patient must repeat pap smear	Disagree	34	42.5	46	57.5	80/85	94.1
If the results come back with persistent LSIL, keep the patient at the clinic without any further action	Disagree	13	17.6	61	82.4	74/85	87.1
The incidence of cervical cancer in our population is very high	Agree	73	94.8	4	5.2	77/85	90.6

ART, antiretroviral therapy; HSIL, high-grade squamous intraepithelial lesion; LSIL, low-grade squamous intraepithelial lesion; HIV, human immunodeficiency virus; WHO, World Health Organization; WLWH, women living with HIV; South African National Department of Health.

† , SA-DOH national cervical cancer screening guidelines were used for the answers.

Most of the HCWs were familiar with cervical cancer screening in WLWH infection in general; however, specific gaps in the knowledge were identified. Some of the identified gaps included lack of knowledge about screening intervals in WLWH, when to refer a patient with an abnormal pap smear or when to repeat a smear.

There was a statistically significant association betweenHCWs and the awareness of the uMgungundlovu District policy (only 2.1% were aware) on cervical cancer screening (*p* = 0.00), and the perception of oneself being compliant with the cervical cancer screening in WLWH (*p* = 0.00). Statistically significant associations were also found between healthcare category and certain Likert scale questions (Table 4).

**TABLE 4:** Association between healthcare workers and variables related to knowledge of guidelines.

Variable	*P*
Are you aware of any cervical cancer screening guidelines?	0.05
Are you aware of the uMgungundlovu District policy on cervical cancer screening?	0.00*
According to your knowledge, do you think your health facility is compliant with the cervical cancer screening of WLWH?	0.00*
All patients with an abnormal pap smear result should be referred for colposcopy.	0.76
All WLWH must have a yearly pap smear.	0.29
A pap smear must be done at the age of 21 years irrespective of HIV status.	0.01*
A pap smear in WLWH is only done after the age of 30 years.	0.00*
Cervical cancer screening in WLWH can be done at anytime.	0.00*
Cervical cancer screening in WLWH can only be done 3 years after initiating ART.	0.49
If the results come back with HSIL, the patient must repeat the pap smear.	0.00*
If the results come back with persistent LSIL, you must keep the patient at the clinic without any further action.	0.59
The incidence of cervical cancer in our population is very high.	0.59

WLWH, women living with HIV; HISL, high-grade squamous intraepithelial lesion; LSIL, low-grade squamous intraepithelial lesion; ART, antiretroviral therapy; HIV, human immunodeficiency virus.

* , Statistically significant association.

## Discussion

To our understanding, this is the first study to evaluate the knowledge and implementation of cervical cancer screening guidelines by HCWs in WLWH in KwaZulu-Natal province. The most crucial finding is that 70% of HCWs knew that screening in WLWH could be done at any age, including at the time of the diagnosis of HIV infection. This is important because this differs from the previous policy that all women should be screened from the age of 30 years onwards. This finding highlights an awareness that WLWH are at a higher risk and should be managed differently. The National Department of Health (NDOH) guidelines were used as the yardstick.

More than 80% of the HCWs agreed that all WLWH must have a yearly pap smear. Current South African recommendations encourage all WLWH who are at increased risk of developing cervical cancer to have yearly pap smears in high-resource settings, and 3 yearly in low-resource settings.^8^ Similarly, Canadian guidelines recommend annual pap tests for WLWH.^9^ One-third of the HCWs agreed that a pap smear should be done at 21 years of age irrespective of HIV status. In this regard, the South African guidelines report that the pap smear must be done at the age of 25 years in HIV-uninfected women whilst in WLWH smears should be done at the time of testing HIV-seropositive.^8^ At present, the NDOH guidelines for the cervical cancer programme offer three cervical cytology smears per lifetime at public health facilities, starting from 30 years, and then at 10-year intervals, in HIV-uninfected women. Guidelines for WLWH include more frequent cytology tests. The National Department of Health recommends screening for WLWH at HIV diagnosis and every 3 years then if screening is normal and yearly if abnormal (LSIL). The WHO advocates at least one smear test (at 30–35 years of age) to be performed in a woman’s lifetime. Women Living with HIV who are < 21 years of age and are sexually active may have a high rate of progression of abnormal cytology.^9^ Brogly and colleagues reported that 30% of adolescents had atypical squamous cells of undetermined significance (ASC-US) or greater on their first cervical pap test.^10^

A small percentage of the HCWs agreed that a pap smear in WLWH is only performed after the age of 30 years, whereas the NDOH guidelines state that in WLWH pap smears should be done at the time of diagnosis of the HIV infection.^8^

More than 40% of the HCWs stated that if the results returned as HSIL, the patient must repeat the pap smear. And more than 17% of HCWs agreed that if the results returned as a persistent LSIL, the patient should be kept at the clinic without any further action. This is not what the guidelines state. It is recommended that for a persistent LSIL or worse (including ASC and atypical glandular cells [AGCs]) and HSIL, women with abnormal pap smears should be referred for a colposcopy assessment.^11,12^

Our study found that HCWs’ responses to cervical screening in WLWH were suboptimal. Most of the HCWs had less than 3 years of experience in initiating ARV drugs and more than 20% had formal training in HIV management, which might have negatively impacted the results.

All the HCWs in our study were aware of the existence of different cervical cancer screening guidelines. In SA, these guidelines are published by the NDOH. Several South African provinces and hospitals have guidelines which they have adapted from the NDOH. It is concerning, however, to note that only 2.1% of KwaZulu-Natal HCWs in this study were familiar with their provincial guidelines. This article recommends that greater attention should be given to the continuing medical education of cervical cancer screening and cervical cancer prevention.

Most of our HCWs were in the early years of their careers. They were aware of both the NDOH and WHO guidelines. The former is used in all South African public sector hospitals and clinics. Although two HCWs answered that there were ‘separate’ KZN guidelines, this was an error.

The study did not find any statistically significant association between the HCWs’ category and their knowledge of the guidelines. This demonstrates the need, regardless of category, for courses in the workplace that target these concerns. On the other hand, significant differences were revealed between HCWs and their awareness of district guidelines or policy, the screening intervals of WLWH and when to refer for colposcopy. Although the majority of participants indicated that their facilities were compliant with the guidelines, the gaps that have been uncovered indicate the need for ongoing education in this regard.

## Limitations

The main limitations of the study are as follows:

- The small number of HCWs: results should be interpreted with caution.
- The use of a questionnaire may not reflect the actual workplace competency of the participants.
- The answers reflect a local or specific context and therefore generalisation of the study results may be limited.
- An operational bias exists in the mixing of different groups of HCWs with different levels of professional experience. Nonetheless and surprisingly, the gaps were widespread and did not link with individual categories of HCWs.

## Conclusion

Healthcare workers were knowledgeable about cervical cancer screening in WLWH but displayed gaps in practice and knowledge. Most of them were well informed about the recommended screening intervals. However, a certain number of HCWs did not know when to refer a patient for further management. A delay in the management of precancerous lesions could lead to morbidity and mortality, particularly in WLWH. Healthcare workers require guidelines customised to local contexts, in particular for the care of WLWH. Recommendations emerging from this study include regular workplace refresher training that highlights the guidelines and related institutional protocols and renewed attention to the monitoring and evaluation of institutional compliance with cervical cancer screening and prevention.
